# Compilation of different data sets of the Late Neolithic wetland site of Pestenacker and of the adjacent valley depositions

**DOI:** 10.1016/j.dib.2022.108481

**Published:** 2022-07-22

**Authors:** Anne Köhler, Anneli Wanger O'Neill, Johannes Rabiger-Völlmer, Franz Herzig, Birgit Schneider, Steven Nebel, Ulrike Werban, Marco Pohle, Manuel Kreck, Peter Dietrich, Lukas Werther, Detlef Gronenborn, Stefanie Berg, Christoph Zielhofer

**Affiliations:** a Institute for Geography, Leipzig University, Leipzig, Germany; b Bavarian State Department for Cultural Heritage (BLfD), Munich, Germany; c Dendroarchaeological Laboratory of Bavarian State Department for Cultural Heritage, Thierhaupten, Germany; d Department Monitoring and Exploration Technologies, Helmholtz Centre for Environmental Research - UFZ, Leipzig, Germany; e Institute for Prehistoric and Medieval Archaeology, University of Tuebingen, Germany; f Römisch-Germanisches Zentralmuseum (RGZM), Leibniz Research Institute for Archaeology, Mainz, Germany

**Keywords:** direct push sensing, driving core drilling, electrical conductivity, Neolithic settlement, southern Germany, Pestenacker

## Abstract

This document contains data sets of the valley depositions of the *Loosbach* valley and data of the Late Neolithic wetland site of Pestenacker. It consists of raw data and graphical figures of direct push-based electrical conductivity and colour logs and driving core recoveries as well as hand drilling recoveries presented by Köhler et al. [1].

We reviewed unpublished archaeological profiles to determine the incision levels of former stream phases at Pestenacker site. Here, we provide the new, reusable and accessible data set.

The data sets and figures of the valley depositions can be used for further analyses, including statistical ones, to improve the methods of the direct-push sensing and to compare it with the sedimentological features recovered from driving core and hand drillings. In addition, the data set is useful for further issues in Pestenacker as well as in the whole central Europe. Especially in the circum-Alpine region, as a comparison with other pile dwellings or stilt houses built from the Neolithic to the Bronce Age.

## Specifications Table

**Table:** 

Subject	Geography
Specific subject area	Characterisation of Holocene valley depositions
Type of data	TableFigure
How data were acquired	Direct push sensing, caterpillar: Geoprobe 6610DTDirect push sensing, electrical conductivity: Geoprobe SC-500Direct push sensing, colour logging tool: Soil Colour Optical Screening Tool (SCOST), Dakota Technologies, Fargo, USADriving core drilling: Cobra Pro, Atlas Copco; with 60 mm diameter open corer (1 m length)Hand drilling: Edelman-Bohrer, Eijkelkamp, with 70 mm diameterSediment features: according to AG Boden [2] and Munsell Soil Colour Chart
Data format	Raw and Analysed
Parameters for data collection	Electrical conductivity was recorded in mS/m. Soil colours were recorded in the Munsell colour system.Coordinates were recorded in UTM (WGS84, zone 32N) format.
Description of data collection	Electrical conductivity was measured with Geoprobe SC 500 probe using a four point Wenner configuration. Soil colour was measured with Soil Optical Color Screening Tool (SCOST™, Dakota Technologies, Fargo, USA) in the wavelength range of 350-1000 nm.
Data source location	Late Neolithic Pestenacker wetland siteCity/Town/Region: Pestenacker, BavariaCountry: GermanyLatitude and longitude (and GPS coordinates, if possible) for collected samples/data: 10.94852-10.94471, 48.14705-48.14532Primary data sources: [3,^4^,5]
Data accessibility	With the articleAndRepository name: Pangaea, Direct Push Sensing with the Soil Optical Color Screening Tool in the Loosbach valley at Pestenacker, a Late Neolithic wetand site, northern Alpine forelands, GermanyName of the data set: Direct Push sensing with the Soil Optical Colour Screening ToolDOI: https://doi.pangaea.de/10.1594/PANGAEA.936097Repository name: Pangaea; Direct Push Electrical Conductivity Sensing in the Loosbach valley at Pestenacker, a Late Neolithic wetand site, northern Alpine forelands, GermanyName of the data set: Direct Push Electrical Conductivity sensingDOI: https://doi.pangaea.de/10.1594/PANGAEA.933520Repository name: Pangaea; Detailed lithological descriptions of recovered cores from the Loosbach valley at Pestenacker, a Late Neolithic wetland site, northern Alpine forelands, GermanyName of the data set: Detailed lithological descriptionsDOI: https://doi.pangaea.de/10.1594/PANGAEA.934386
Related research article	**Or, for a co-submission (when your related research article has not yet published):**A. Köhler, A. Wanger-O'Neill, J. Rabiger-Völlmer, F. Herzig, B. Schneider, S. Nebel, U. Werban, M. Pohle, M. Kreck, P. Dietrich, L. Werther, D. Gronenborn, S. Berg, C. Zielhofer, A hydrological tipping point and onset of Neolithic wetland occupation in Pestenacker (Lech catchment, S Germany). Quat. Sci. Rev. In Press.

## Value of the Data

• The presented data set of the Holocene valley depositions are useful for reconstructing environmental conditions.
• The data is interesting for scientist working in the fields of Holocene sedimentology in the circum-Alpine region, Late Neolithic Archaeology.
• The data of the valley depositions can be used for further statistical verification of the two direct push methods (electrical conductivity sensing, Soil Colour Optical Screening Tool). The data can be further analysed and compared with other archaeological sites.

## 1 Data Description

Fig. 1a and 1b shows the positions of the direct push sensing and the drilling cores at the Late Neolithic wetland site of Pestenacker in the valley of the Loosbach. The detailed raw data of the coordinates are accessible in the data repository [3,^4^,5].

**Fig. 1a:**
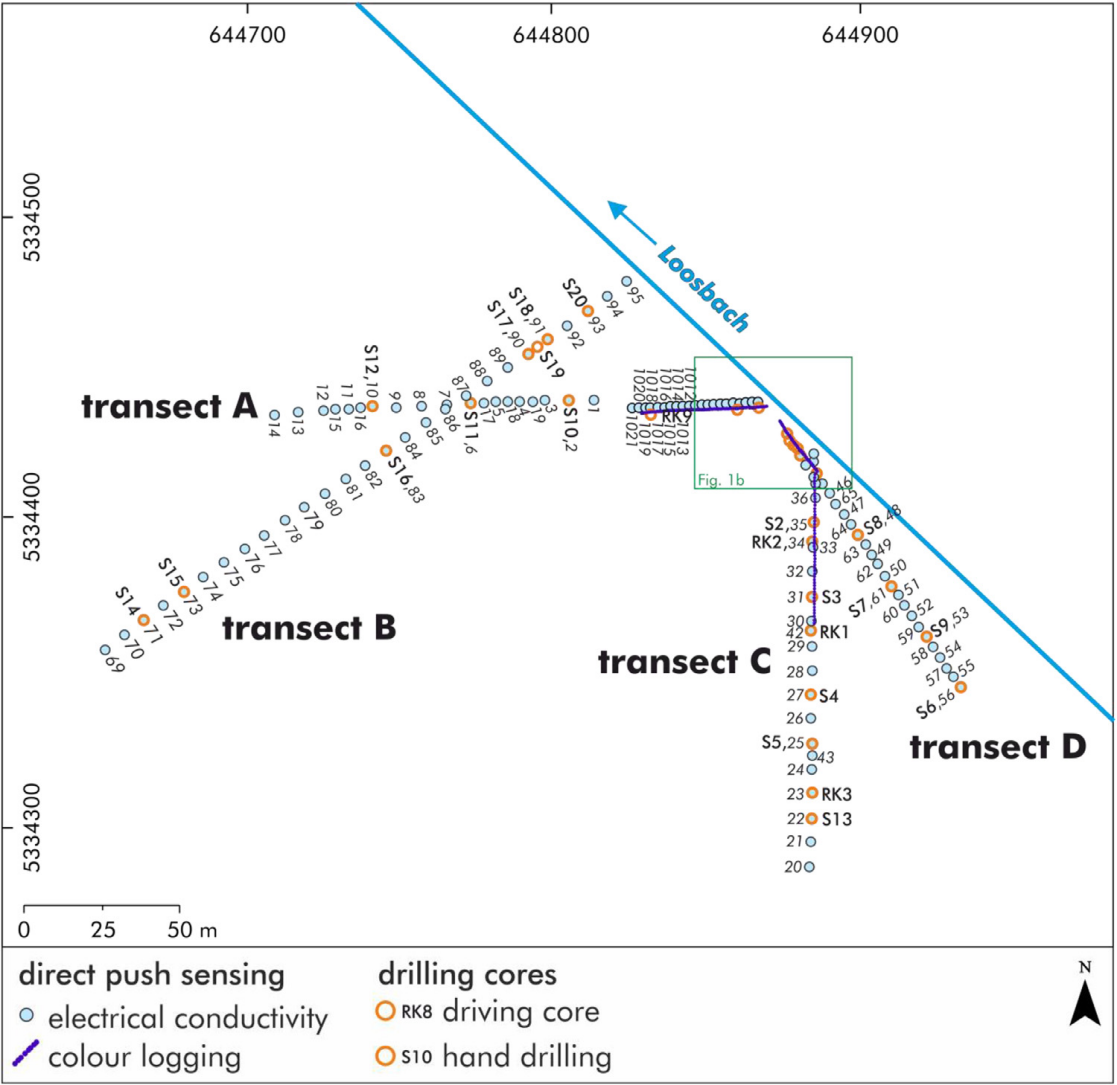
Study area with drilling and direct push sensing positions. Modified from Köhler et al. [1]Fig. 3.

**Fig. 1b:**
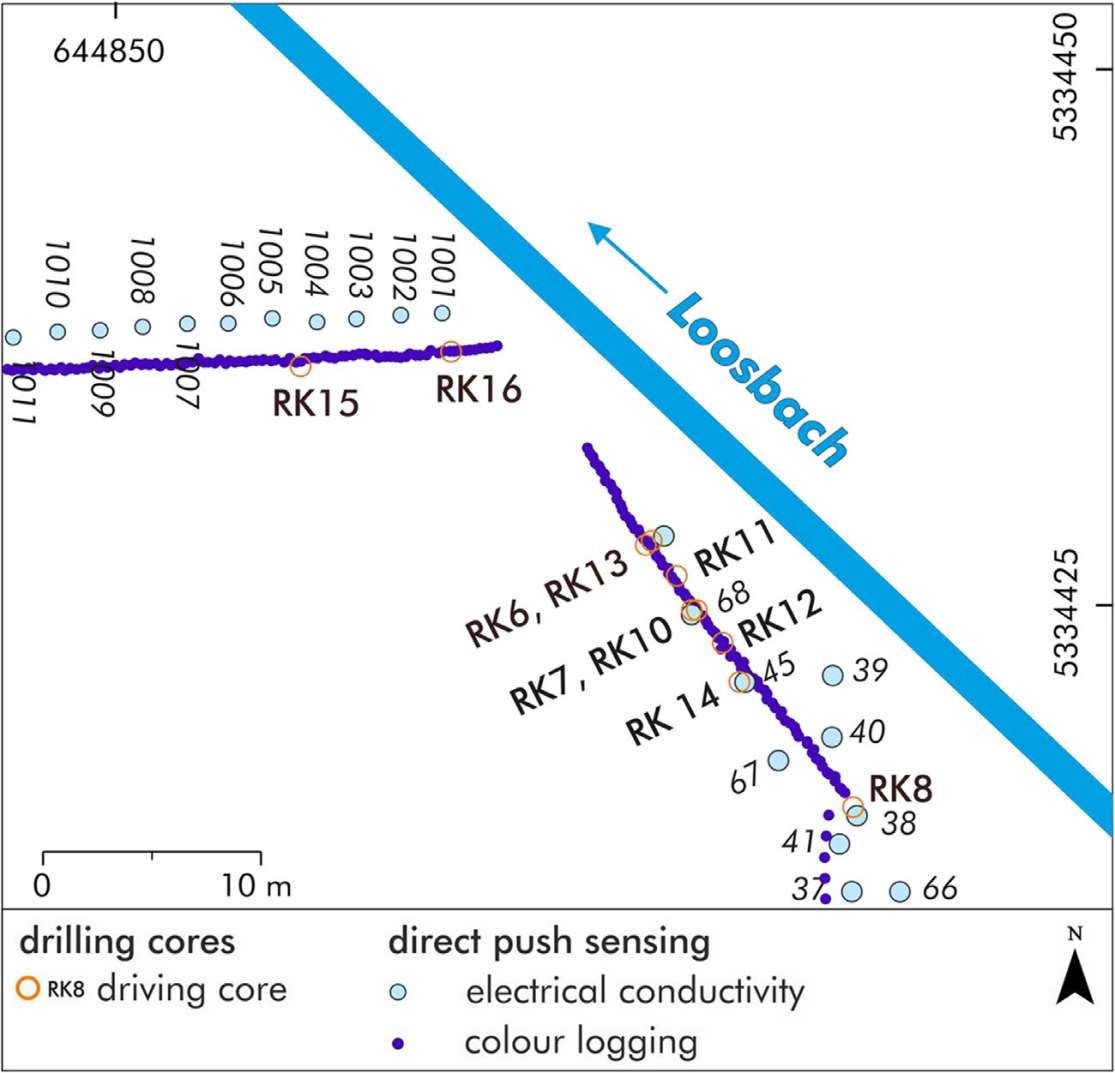
Detailed study area with drilling and direct push sensing positions. Modified from Köhler et al. [1]Fig. 4.

**Fig. 2:**
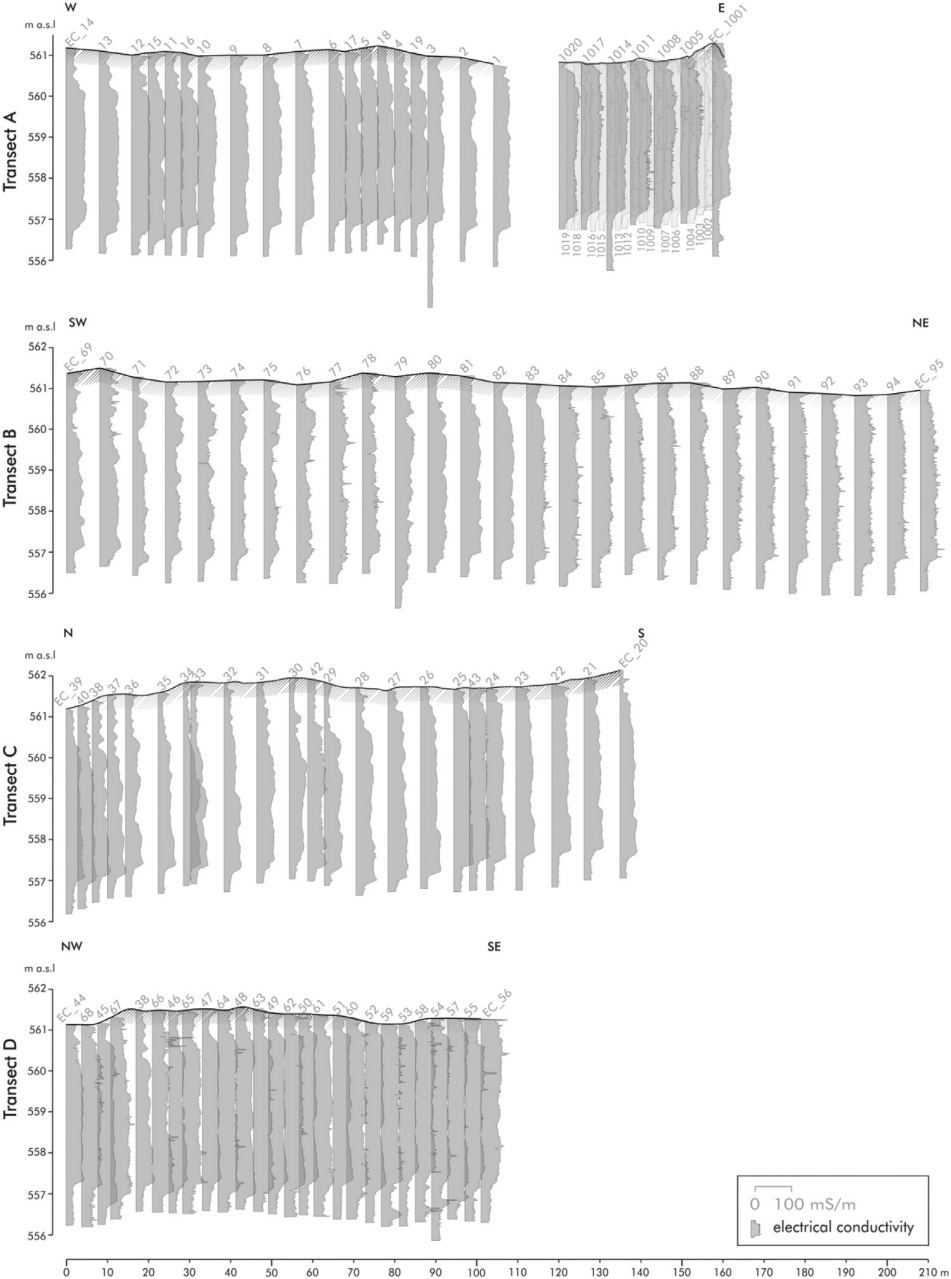
Direct push electrical conductivity sensing of transects A to D. Modified from Köhler et al. [1] Figs. 6 and 7.

Fig. 2 represents the data of the direct push electrical conductivity sensing of transect A to D. The individual logs are adapted to the respective profile line. The numbers above stand for the respective log numbers. Most of the logs are around 5 m deep. Only individual ones were pushed deeper (e.g., EC_3, EC_79, EC_1014).

Transect A (west-east orientation) consists of 39 logs over a total length of 160 m. The southwest-northeast transect (transect B) includes 27 logs with an almost equidistant distance of approx. 8 m. Transect C, orientated from north to south, contain 23 electrical conductivity logs. The last transect D (from northwest to southeast) consists of 26 logs with an equidistant distance of approx. 4 m. The detailed raw data of the direct push electrical conductivity, e.g., the electrical conductivity with the respective depth of measurement below surface, are accessible in the data repository [3]. This data includes the sample label, the exact location (latitude, longitude, altitude), the depth of the measurements (in m below surface) and the related electrical conductivity measured in mS/m.

Fig. 3a and 3b show the lithological descriptions of the 14 driving cores and 20 hand drillings of transect A to D. Fig. 3b shows the detailed section of the north-western part of transect D. The abbreviations above stand for the respective names of the drilling cores. An ‘S’ stands for hand-drilling and ‘RK’ represents driving core drillings. The sedimentological findings were classified into eight stratigraphical units: stratigraphical gap (white); precipitated carbonate, humic and overbank deposits (pale blue); very dark brown to black organic-rich layer (dark brown); loamy grey layer (dark grey); archaeological layer (pale pink); transition from archaeological layer to freshwater carbonates (beige with pale pink diagonal stripes); freshwater carbonates (beige); peat (brown); gravel (pale grey with dark grey ovals). Certain features (e.g., charcoal, wood remains, brick fragments) were also shown. With a depth of four to five metres, the driving cores are deeper the hand drillings. Gravels were identified at a depth of approx. four meters. Above the gravels there are alternating layers of peat and freshwater carbonates. Six cores are shown on transect A (west to east). Archaeological remains were discovered in the eastern part. Transect B (from southwest to northeast) includes seven hand drillings. A total of four driving cores and five hand drillings are located at the north-south transect (C). The most drilling cores on a transect occur on the last transect D, especially in the north-western part with a lot of archaeological remains. In this part there are eight drilling cores on 16 m. In the south-eastern part there are three hand drilling every 20 m. The detailed raw data of the lithological descriptions are accessible in the data repository [4]). The data includes the drillings (column A) with the exact location (longitude, latitude, altitude in columns B-D) and the description of the individual cores used for documentation and analyses (only for driving cores, measured in centimetres) in column E. Columns F-G indicates the depth of layers below the surface and columns H-I the respective depths adapted to the real height above sea level in metres. The depths of the sediment samples used for geochemical analyses are shown in column J (measured in cm below surface). The name of the stratigraphical unit, classified according to the stratigraphical and geochemical findings, are listed in column K. Column L show the colour according to “Munsell Soil Colour Chart”. The estimated carbonate content according to AG Boden ([2], Tab. 40, p. 169) is shown in column M. Column N includes further information and details about the layers and column O the detailed description of the carbonate concretions. The remarks about grain size of the siliciclastic fine soil, literally or according to AG Boden ([2], pp. 141) are listed in column Q. Information about further (no sediment) samples (the name and the material) can be found in columns R-S.

**Fig. 3a:**
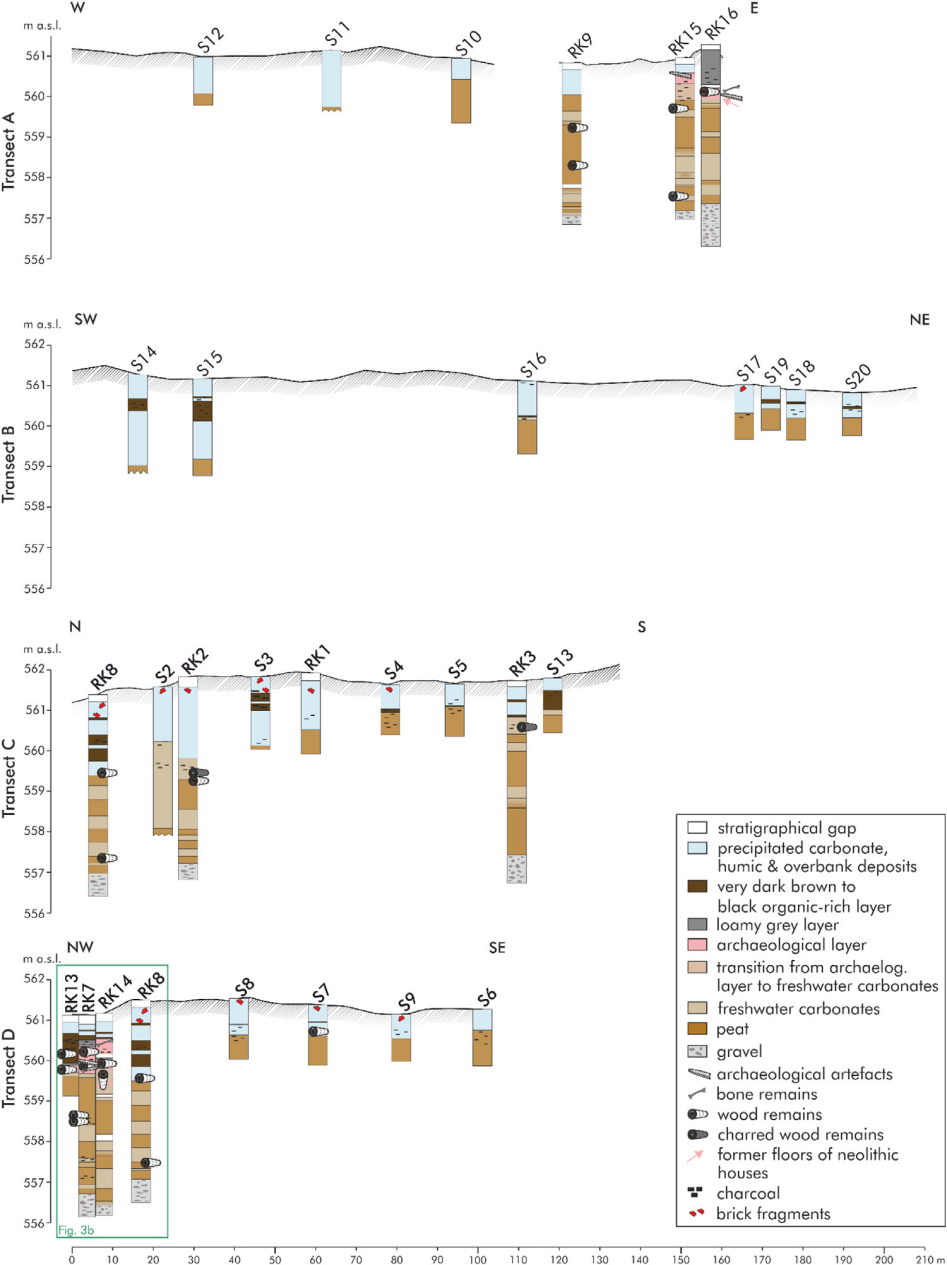
Data of driving cores and hand drillings of transect A to D. Modified from Köhler et al. [1] Figs. 6 and 7.

**Fig. 3b:**
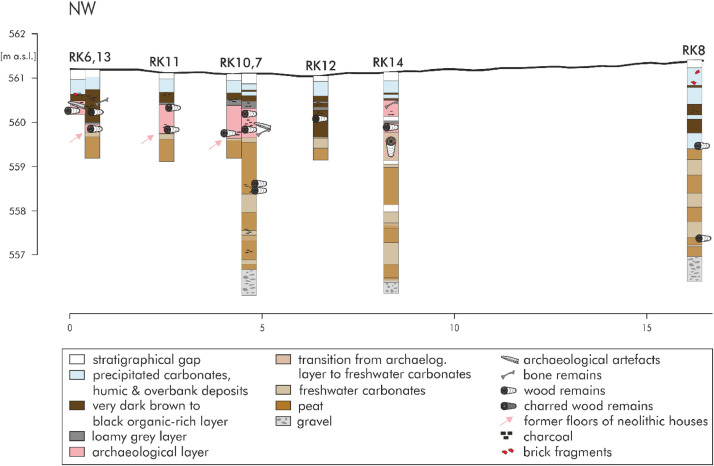
Data of driving cores of the northern part of transect D. Modified from Köhler et al. [1] Fig. 7.

**Fig. 4:**
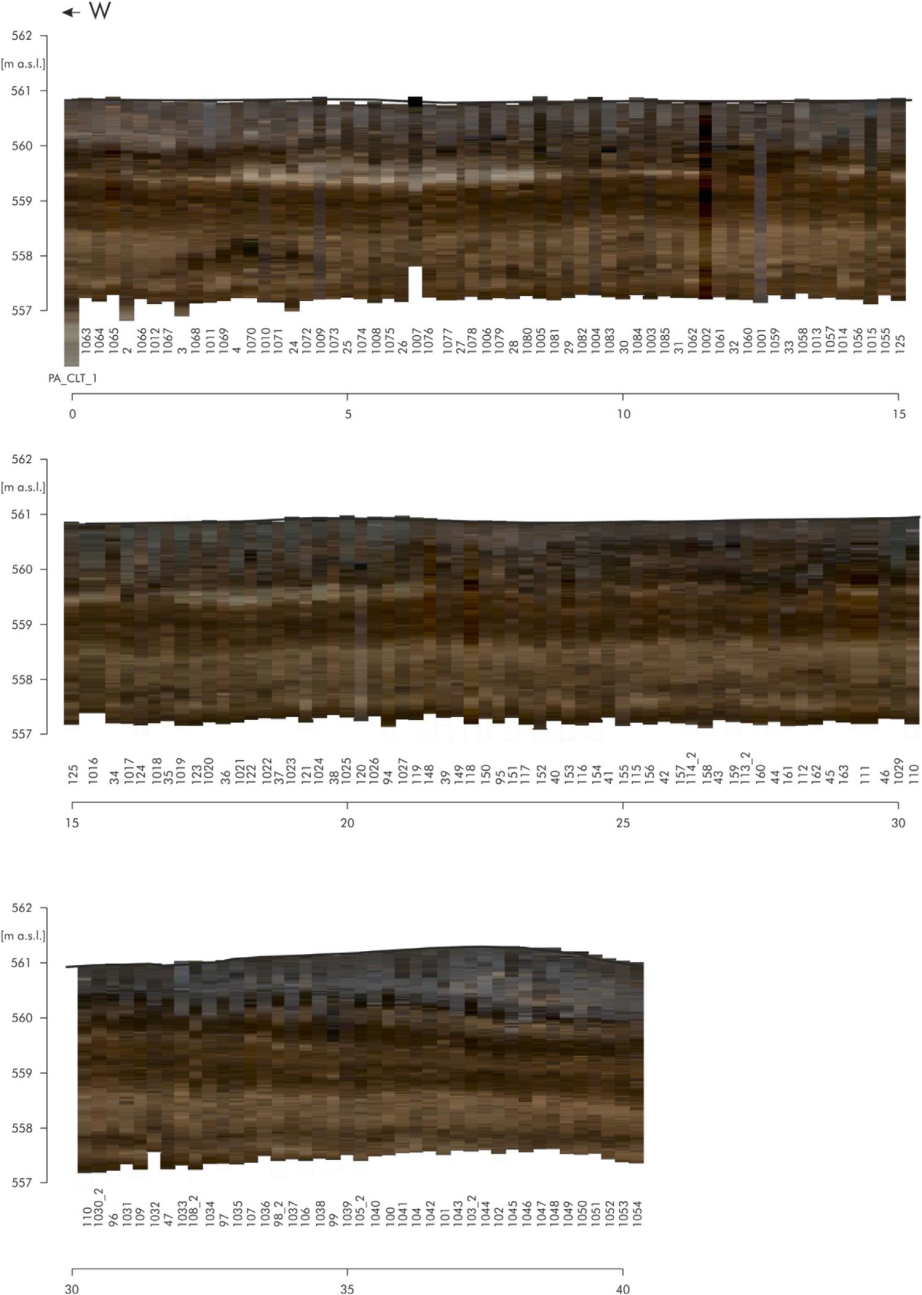
RGB Raster data of the direct push colour logs of transect A. Modified from Köhler et al. [1] Fig. 6.

**Fig. 5:**
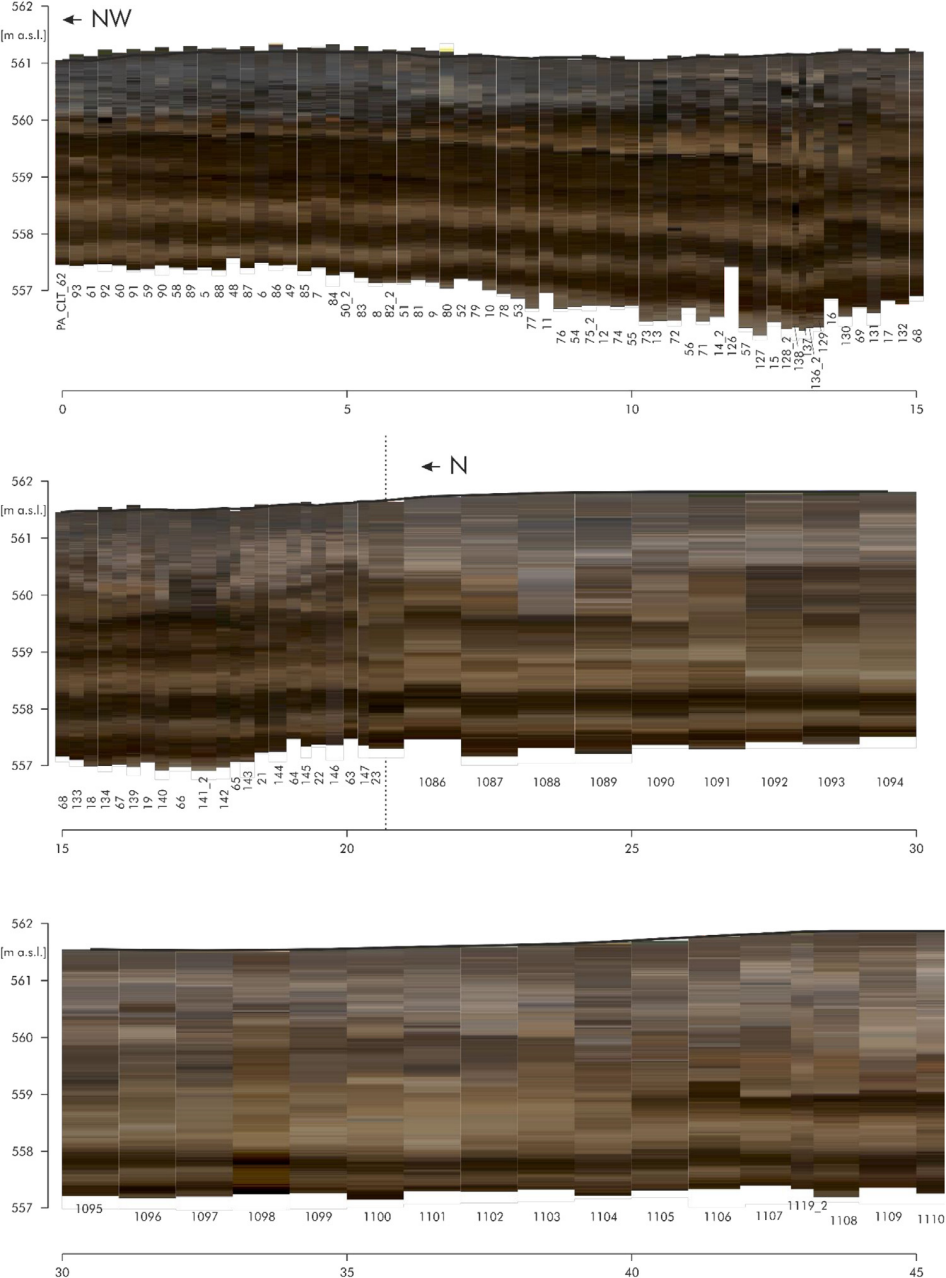

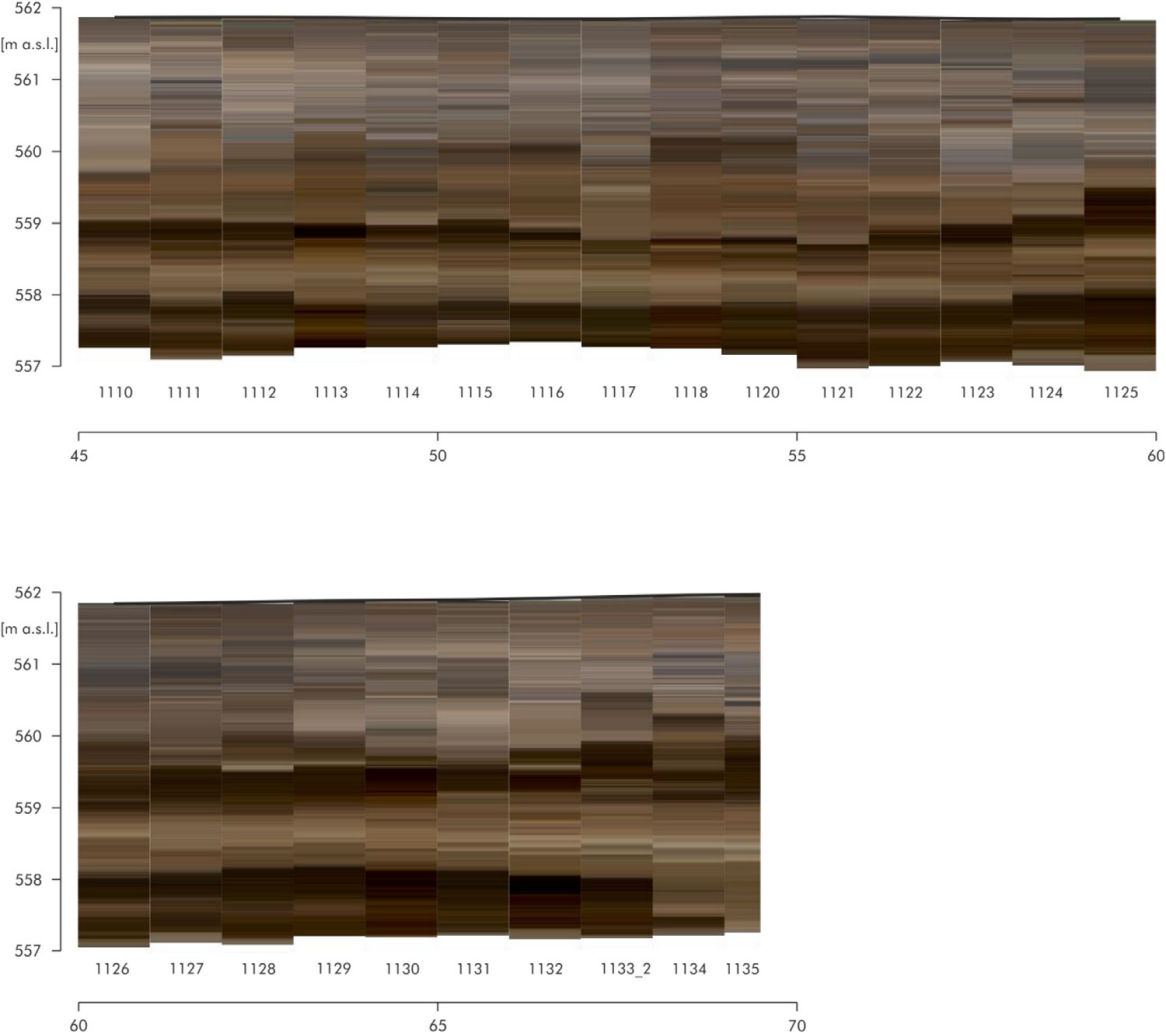
RGB Raster data of the direct push colour logs of transect C and D. Modified from Köhler et al. [1] Fig. 7.

Fig. 4 shows the RGB raster data of the direct push colour logs of the eastern part of transect A. The individual logs are adapted to the respective profile line. The numbers below stand for the respective log numbers. The logs are located at an equidistant distance of approx. 25 cm. There are a total of 161 logs each with a depth of approx. four meters over a distance of 60 m. Fig. 5 shows the RGB raster data of the direct push colour logs of the northern part of transect C and the north-western part of transect D. The dashed line at log ‘PA_CLT_23’ points to the intersection of these two transects. The individual logs are adapted to the respective profile line. The numbers below stand for the respective log numbers. The north-western part consists of 86 colour logs located at an equidistant distance of approx. 25 cm. The other logs (n=50) are also equidistant but with a distance of approx. one meter. The raw data of the direct push colour logs (detailed coordinates and colours in the Munsell colour system with the corresponding depths) are accessible in the data repository [5]. This data includes the names of the logs (event), the exact location (longitude, latitude, altitude), the depth of the measurements (in m below surface) and the related colour according to the Munsell Soil Colour Chart (colour HLS, value and chroma).

Table 1 compiles the incisions levels of former stream courses of the Loosbach. It shows the values at four different time intervals generated from unpublished archaeological excavation profiles. The column “ID” represents the internal ID of archaeological excavation profiles. The quadrant (according to the excavations reports and maps) and the direction of the excavation profiles are documented in the column “quadrant” and “direction”. The individual incision levels of the different intervals are shown in the columns “during settlement phases”, “at the end and after the settlement”, “during the Middle Ages” and “Loosbach between 1868-1934”. Settlement represents the Late Neolithic wetland site of Pestenacker.

**Table 1:** Incision levels of former stream courses of the *Loosbach*

			incision levels of former streams [m a.s.l]			
ID	quadrant	direction	during settlement phases (IL2)	at the end and after settlement phases (IL3)	during the Middle Ages (IL5)	Loosbach between 1846-1934 (IL6)
17551756	C IV	south				560.35
17611762	C V	east				560.11560.41
16701671	D IV	north				560.75
16661667	D IV	east			560.4	
16681669	D IV	west			560.05560.25	560.4
17741775	E IV	north	559.72			
17721773	E IV	south	559.11			
17811782	F III	north	559.78			
178317841785	FIII	west				560.4
17921793	F IV	east	559.04			
1830	G VI	west		559.25		
18261827	G VI	south		559.42		
18361937	G VII	south		559.8		
18461847	H IV	south		559.35		
18601861	H V	west		559.45		
185518581859	H V	north		559.4		
18561857	H V	east		559.35		
18531854	H V	south		559.45		
186418651866	H VI	south		559.83		
18711872	J I	north		559.15		
1874	J I	east		559.3		
190119021903	J V	east		559.64		
19211922	K I	north			559.75	560.05

## 2 Experimental Design, Materials and Methods

The compiled data in this document was generated using different methods. The study area of the Late Neolithic wetland site of Pestenacker is situated at the edge of the *Loosbach* valley, a tributary valley of the *Lech* River, approx. 15 km northeast of Landsberg am Lech. The detailed maps are shown by Köhler et al. [1].

For the data of the Holocene depositions of the Loosbach valley at Pestenacker, an UNESCO world heritage site of Late Neolithic wetland occupation, 297 direct push colour logs and 116 direct push electrical conductivity logs were conducted. The direct push sensing of depth-accurate electrical conductivity was performed by self-propelled carrier vehicle (Geoprobe) with attached Geoprobe SC 500 probe using a four point Wenner configuration. The Soil Optical Color Screening Tool (SCOST™, Dakota Technologies, Fargo, USA) was applied for sensing of visible colours [6,^7^,8]. This colour logging tool (CLT) induces white light and records the reflected light from the sediment in the wavelength range of 350-1000 nm. Raster images, the corresponding colour of the Munsell colour system and depth information are computed by the OST-Software (Dakota Technologies, Fargo, USA) [7,8]. The depth resolution depends on the probing velocity and is in this study in cm-scale. The logs were terminated, when the resistance increased. All of the 332 direct push soundings were levelled by a Topcon HiPer II DGPS system in cm-resolution. The detailed raw data of the direct push logs are accessible in the data repository [3,5].

The 14 driving cores and 20 hand drillings were carried out to generate the data of the Holocene depositions of the Loosbach valley at Pestenacker, an UNESCO world heritage site of Late Neolithic wetland occupation. A hand-held Cobra Pro (Atlas Copco) driving core drilling system and 60 mm diameter open corer recovered sediments. with 70 mm diameter. The achieved segments of each 1 m lengths were accessed on site towards sediment features and colours according to [2] and Munsell Soil Colour Chart. For hand drilling the Edelman-Bohrer (Eijkelkamp) with a diameter of 70 mm was used. The following parameters were recovered: colour (according to the Munsell Soil Colour Chart), the estimated carbonate content and the grain size of the fine soil (according to [2]), redox features, detailed descriptions about the carbonate concretions and further remarks and details about the layer. The sedimentological findings were classified into eight stratigraphical units. The raw data of the detailed lithological descriptions are accessible in the data repository [4].

The incision levels of former stream courses of the *Loosbach* were generated using unpublished excavation reports. In addition, we assigned radiocarbon, archaeological and historic age controls to the different stream courses, which enables the development of the stream courses in six different time intervals. The respective age data are shown in Köhler et al. [1].

## Ethic Statements

We confirm that the manuscript adheres to the standards of ethics in publishing.

## CRediT Author Statement

**Anne Köhler:** Conceptualization, Methodology, Formal analysis, Investigations, Data curation, Writing – original draft, Visualization, Writing – review & editing; **Anneli Wanger-O'Neill:** Formal analysis, Data curation, Methodology, Writing – review & editing; **Johannes Rabiger-Völlmer:** Formal analysis, Investigations, Data curation, Writing – review & editing; **Franz Herzig:** Investigations, Resources, Writing – review & editing; **Birgit Schneider:** Formal analysis, Resources, Data curation; **Steven Nebel:** Investigations; **Ulrike Werban:** Methodology, Writing – review & editing; **Marco Pohle:** Formal analysis, Investigations, Data curation; **Manuel Kreck:** Formal analysis, Investigations, Data curation; **Peter Dietrich:** Methodology, Resources, Funding acquisition; **Lukas Werther:** Methodology, Writing – review & editing, Funding acquisition; **Detlef Gronenborn:** Writing – review & editing; **Stefanie Berg:** Methodology, Writing – review & editing, Funding acquisition; **Christoph Zielhofer:** Conceptualization, Methodology, Investigations, Writing – original draft, Writing – review & editing, Supervision, Funding acquisition.

## Declaration of Competing Interest

The authors declare that they have no known competing financial interests or personal relationships which have or could be perceived to have influenced the work reported in this article.

## Data Availability

Direct Push Electrical Conductivity Sensing in the Loosbach valley at Pestenacker, a Late Neolithic wetand site, northern Alpine forelands, Germany (Original data) (PANGAEA). Detailed lithological descriptions of recovered cores from the Loosbach valley at Pestenacker, a Late Neolithic wetland site, northern Alpine forelands, Germany (Original data) (PANGAEA). Direct Push Sensing with the Soil Optical Color Screening Tool in the Loosbach valley at Pestenacker, a Late Neolithic wetand site, northern Alpine forelands, Germany (Original data) (PANGAEA).
